# Applications of functional data analysis: A systematic review

**DOI:** 10.1186/1471-2288-13-43

**Published:** 2013-03-19

**Authors:** Shahid Ullah, Caroline F Finch

**Affiliations:** 1 Flinders Centre for Epidemiology and Biostatistics, School of Medicine, Faculty of Health Sciences, Flinders University, Adelaide, SA, 5001, Australia; 2 Centre for Healthy and Safe Sports (CHASS), University of Ballarat, SMB Campus, Ballarat, VIC, 3353, Australia

**Keywords:** Functional data analysis, Smoothing, Functional principal component analysis, Clustering, Functional linear model, Forecasting, Time series data

## Abstract

**Background:**

Functional data analysis (FDA) is increasingly being used to better analyze, model and predict time series data. Key aspects of FDA include the choice of smoothing technique, data reduction, adjustment for clustering, functional linear modeling and forecasting methods.

**Methods:**

A systematic review using 11 electronic databases was conducted to identify FDA application studies published in the peer-review literature during 1995–2010. Papers reporting methodological considerations only were excluded, as were non-English articles.

**Results:**

In total, 84 FDA application articles were identified; 75.0% of the reviewed articles have been published since 2005. Application of FDA has appeared in a large number of publications across various fields of sciences; the majority is related to biomedicine applications (21.4%). Overall, 72 studies (85.7%) provided information about the type of smoothing techniques used, with B-spline smoothing (29.8%) being the most popular. Functional principal component analysis (FPCA) for extracting information from functional data was reported in 51 (60.7%) studies. One-quarter (25.0%) of the published studies used functional linear models to describe relationships between explanatory and outcome variables and only 8.3% used FDA for forecasting time series data.

**Conclusions:**

Despite its clear benefits for analyzing time series data, full appreciation of the key features and value of FDA have been limited to date, though the applications show its relevance to many public health and biomedical problems. Wider application of FDA to all studies involving correlated measurements should allow better modeling of, and predictions from, such data in the future especially as FDA makes no *a priori* age and time effects assumptions.

## Background

Recent increased interest in the application of statistical modeling to medicine, biomedicine, public health, biology, biomechanics and environmental science has largely been driven by the need for good data to inform government policy and planning processes for health service delivery and disease prevention. Importantly, such models will only be useful in the long term if they are accurate, based on good quality data, and generated through application of robust appropriate statistical methods. Functional data analysis (FDA) is one such approach towards modeling time series data that has started to receive attention in the literature, particularly in terms of its public health and biomedical applications.

Commonly, time series data are treated as multivariate data because they are given as a finite discrete time series. This usual multivariate approach completely ignores important information about the smooth functional behavior of the generating process that underpins the data [1]. It also suffers from issues associated with highly correlated measurements within each functional object. The basic idea behind FDA is to express discrete observations arising from time series in the form of a function (to create functional data) that represents the entire measured function as a single observation, and then to draw modeling and/or prediction information from a collection of functional data by applying statistical concepts from multivariate data analysis. In doing so, it has the advantage of generating models that can be described by continuous smooth dynamics, which then allow for accurate estimates of parameters for use in the analysis phase, effective data noise reduction through curve smoothing, and applicability to data with irregular time sampling schedules. Ramsay [2,3] presents a strong argument for FDA.

Ramsay and Dalzell [4] present several practical reasons for considering functional data:

1) smoothing and interpolation procedures can yield functional representations of a finite set of observations;

2) it is more natural to think through modeling problems in a functional form; and

3) the objectives of an analysis can be functional in nature, as would be the case if finite data were used to estimate an entire function, its derivatives, or the values of other functionals.

Müller has recently described important characteristics of FDA [5]. The FDA approach is highly flexible in the sense that the timing intervals for data observations do not have to be equally spaced for all cases and can vary across cases. Importantly, FDA methods are not necessarily based on the assumption that the values observed at different times for a single subject are independent. Although functional data themselves are not new, a new conceptualization of them has become necessary because of the increasing sophistication of available data collections [4]. Data collection technology has evolved over recent decades, allowing more dense sampling of observations over time, space, and other continuum measures. Such data are usually interpreted as reflecting the influence of certain smooth functions that are assumed to underlie and to generate the observations. Although classical multivariate statistical techniques are often applied to such data, they do not take advantage of additional information that could be implied by the smoothness of underlying functions. In particular, FDA methods can often extract additional information contained in the function and its derivatives [6,7] that is not normally available from application of traditional statistical methods [1]. Because the FDA approach essentially treats the whole curve as a single entity, there is also no concern about correlations between repeated measurements. This represents a change in philosophy towards the handling of time series and correlated data [8].

There are a number of good illustrations of applications of FDA; for example, Ramsay and Silverman [9,10] using curves as data, Locantore et al. [11] with images as data, and Yushkevich and Pizer [12] where the data points are shape representations of body parts. Application of FDA has also been published across various scientific fields including analysis of child size evolution [9], climatic variation [4,13], handwriting in Chinese [14], acidification processes [15], land usage prediction based on satellite images [16], medical research [17-19], behavioral sciences [20], term-structured yield curves [21], and spectrometry data [22]. Most recently, Ullah and Finch [23] found FDA to be an effective exploratory and modeling technique for highlighting trends and variations in the shape of the age–falls injury incidence relationship over time.

In contrast to most other methods commonly used to model trends in time series data, a key strength of the FDA approach is that it makes no parametric assumptions about age or time effects. The FDA methods for modeling and forecasting data across a range of health and demographic issues also have significant advantages for better understanding trends, risk factor relationships, and the effectiveness of preventive measures [24,25]. In the book *Functional Data Analysis*, Ramsay and Silverman [9] give an accessible overview of the foundations and applications of FDA. In an earlier book entitled *Applied Functional Data Analysis*, the same authors [10] provide many examples that share the property of being functional forms of a continuous variable, most often age or time. In 2004, *Statistica Sinica* published a special issue that included two relevant review articles that dealt exclusively with the close connection between longitudinal data and functional data [26,27]. In his PhD thesis, Ullah [28] described the significance and application of FDA in demographic data settings. Software developed for MATLAB, S-PLUS and R by Ramsay and Silverman specifically to support FDA is available from <http://www.psych.mcgill.ca/misc/fda/>.

Because the application of FDA is still relatively novel, especially to public health and biomedical data, this paper reviews applications of the approach to date with the aim of encouraging researchers to adopt FDA in future studies. This paper begins with a systematic review of the focus and application features of published peer-reviewed FDA studies. In doing so, it provides a summary of the extent to which FDA has been applied in different fields, including an overview of the nature of the time series variables/data used. For each of the identified studies, this paper also describes the features of FDA that were used, including the:

(1) representation of data via principal components analysis, which plays a key role in defining smoothness and continuity conditions of the resulting data;

(2) classification of data, which produces different functional groups (or clusters) for gaining more sophisticated knowledge of different pathways and/or functions for large scale data;

(3) functional linear models used for testing the effects on outcomes in functional form; and

(4) forecasting via stochastic methods, to measure the forecast uncertainty through the estimation of a prediction interval.

## Methods

This review was conducted according to the Preferred Reporting Items for Systematic Reviews and Meta-Analyses (PRISMA) Statement [29]. We conducted a systematic search of 11 electronic databases to identify peer-reviewed FDA application studies published between January 1995 and December 2010. The databases used were Academic Search Premier, ScienceDirect, SpringerLink, Cambridge Journals, MEDGE (Informit), Oxford Journals, PubMed, Sage Journals Online, Web of Science, Wiley Interscience Journals, and MEDLINE. We used the phrase *functional data analysis* to identify relevant articles*,* and considered only English language articles published in peer-reviewed journals. In addition to the electronic database search, the search strategy included secondary searching of the reference lists of identified articles.

### Inclusion and exclusion criteria

Studies were eligible for inclusion if they were original research articles in peer-reviewed journals reporting an application of FDA. We excluded studies of statistical methodology development without application, and abstracts, letters, and conference papers.

### Identification of studies

The first author, with the assistance of two research assistants, sourced and screened all identified articles. This included viewing titles and reading abstracts. We obtained full text versions of potentially eligible articles, assessed them against the exclusion/inclusion criteria, and removed obvious exclusions.

**Figure 1:**
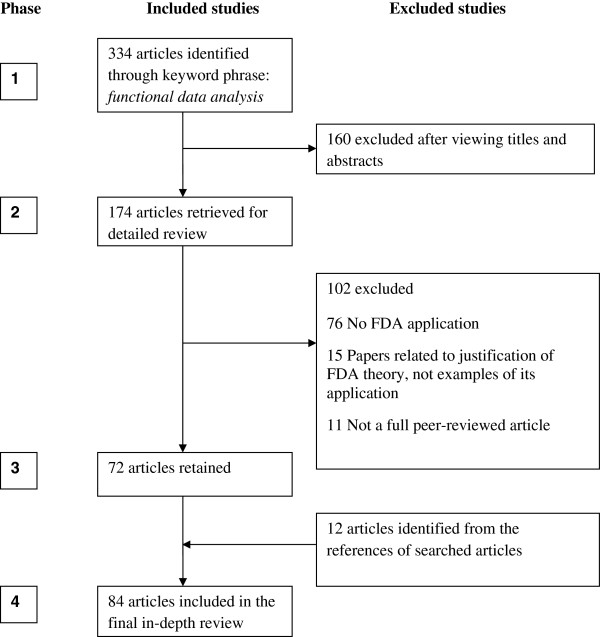
Systematic search strategy used to identify 84 peer-review studies with published application of functional data analysis (FDA).

In the first review phase, 334 articles were identified. Figure 1 summarizes the numbers of studies identified and the reasons for exclusion at each stage. Searching the titles and abstracts of identified studies excluded 160 (47.9%) articles that were not directly relevant to statistical FDA applications. These included reports of functional magnetic resonance imaging (fMRI) to assess patterns of brain activation in patients suffering from chronic traumatic brain injury [30], functional performance in participants with functional ankle instability [31], and the relationship between neurocognitive function and noncontact anterior cruciate ligament injuries [32].

In the second phase, we conducted a complete detailed review of the remaining 174 retrieved articles to ensure they fully met the inclusion and exclusion criteria. A further 102 articles were excluded at this stage, leaving 72 peer reviewed articles for the third phase. Studies excluded at this stage were mainly those that justified FDA theory rather than presenting examples of its application [8,33-35]. A further 12 articles were found in the manual search of reference lists of the 72 retained articles.

We retained a final set of 84 articles for detailed review. The lead author reviewed each paper in terms of key FDA criteria, as outlined below, and assessed its field of application and the specific FDA methods applied. Figure 1 uses the PRISMA [29] flowchart to summarize all stages of the paper selection process.

## Results

### Overview of the published FDA studies

Table 1 summarizes the final set of reviewed papers, and shows fields of application, outcome of interest, and use of the following important FDA features:

• smoothing technique;

• use of functional principal component analysis (FPCA);

• type of clustering adjustment;

• functional linear modeling (FLM) approach adopted to relate explanatory and outcome variables; and

• type of forecasting (if any).

**Table 1:** Areas of application and the functional data analysis (FDA) features used in the 84 peer-review papers reporting application of FDA

**Year**	**Field of study**	**Outcome of interest**	**FDA features**					**Reference**
			**Smoothing**	**Data reduction**	**Clustering**	**FLM**	**Forecasting**	
2010	Biomechanics	Walking velocity on force platform	-	-	-	FRM	-	[36]
	Biomechanics	Kinematic gait data	Polynomial spline	FPCA	-	-	-	[37]
	Biomedicine	Diffusion tensor imaging fiber images	Kernel	-	-	FRM	-	[38]
	Biomedicine	Gene expression microarray data	Local polynomial	FPCA	FEM	-	-	[39]
	Biomedicine	Spinal cord dorsal horn neurons	Locally weighted regression (LOESS)	-	K-Means	-	-	[40]
	Demography	Age-specific mortality rates	Kernel	FPCA	-	-	-	[41]
	Environment	Gas emissions	Kernel	-	-	-	-	[42]
	Geophysics	Magnetometer	Kernel	FPCA	-	FRM	-	[43]
	Medicine	Human growth	-	FPCA	-	-	-	[44]
	Medicine	Age-specific breast cancer mortality rates	Penalized regression spline	FPCA	-	-	State space model	[45]
	Medicine	Age-specific fall injury incidence rates	Penalized regression spline	FPCA	-	-	State space model	[23]
	Medicine	Human vision	Wavelet	-	-	FANOVA	-	[46]
2009	Biology	Temporal fertility trajectories of medfly	-	FPCA	-	FMANOVA	-	[47]
	Biomechanics	Kinematic gait data	-	-	-	FRM	-	[48]
	Biomedicine	3-Tesla magnetic resonance imaging data	-	FPCA	-	-	-	[49]
	Biomedicine	Denaturing gradient gel electrophoresis data	B-spline	FPCA	HCA	-	-	[50]
	Biomedicine	microRNA transfection time-series microarray expression images	B-spline	FPCA	-	-	-	[51]
	Biomedicine	Paediatric diffusion tensor imaging images	B-spline	FPCA	-	-	-	[52]
	Biomedicine	Positron emission tomography time course data	Local polynomial	FPCA	-	-	-	[53]
	Meteorology	Clickstream web data (Hurricane Katrina)	B-spline	-	-	FANOVA	-	[54]
2008	Biomechanics	Ankle dorsiflexion, knee flexion, Achilles tendon, calcaneal and leg abduction angles	Roughness penalty	FPCA	-	-	-	[55]
	Biomedicine	Colon carcinogenesis experiments	Regression splines	FPCA	-	-	-	[56]
	Biomedicine	Diffusion tensor imaging fiber images	B-spline	FPCA	-	-	-	[52]
	Biomedicine	Temporal gene expression profiles for the Drosophila life cycle	Smoothing spline	FPCA	-	FRM	-	[57]
	Biomedicine	Time-course gene expression data	B-spline	-	SVM	-	-	[58]
	Biomedicine	Time-course gene expression data	B-spline	FPCA	LDA, QDA KNN, SVM	-	-	[59]
	Demography	Mortality, fertility and migration rates	Weighted penalized regression spline	FPCA	-	-	State space model	[60]
	Ecology	Plankton monitoring data	Roughness penalty	FPCA	-	-	-	[61]
	Environment	Diurnal ozone and NOx cycles for transportation emission control	Fourier	FPCA	HCA	-	-	[62]
	Finance	Cash flow and transactions	Wavelet	FPCA	-	-	FAR	[63]
	Finance	Price formation and online auctions	Polynomial spline	-	-	FRM	-	[64]
	Linguistics	Speech production variability in fricatives of children and adults	B-spline	FPCA	-	-	-	[65]
	Meteorology	Plasma biomarkers	Kernel	-	-	FRM	-	[66]
	Psychology	Emotional responses of musical listeners	Cubic B-spline	-	-	FANOVA	-	[67]
2007	Biology	Time-course gene expression yeast cell cycle	B-spline	FPCA	MBC	-	-	[68]
	Biomedicine	MRI images	B-spline	-	-	-	-	[69]
	Demography	Mortality and fertility rate	Penalized regression spline	FPCA	-	-	State space model	[25]
	Engineering	Radar waveforms	Kernel	-	HCA	-	-	[70]
	Environment	Diurnal ozone/NOx cycles and transportation emissions	Fourier	-	-	FANOVA	-	[71]
	Environment	Stratospheric ozone levels	Cubic spline	FPCA	-	-	-	[72]
	Medicine	Age-specific breast cancer mortality rates	Weighted local quadratic	FPCA	-	-	State space model	[24]
	Medicine	Women urinary hormone profiles at midlife	Cubic spline	FPCA	-	-	-	[73]
	Medicine	Haemoglobin levels in renal anaemia	B-spline	-	-	-	-	[74]
	Neurology	Joint coordination data in motor development	B-spline	FPCA	-	-	-	[75]
2006	Biology	Time-course gene expression yeast cell cycle	B-spline	FPCA	FLR	-	-	[76]
	Behavioural	Male medfly calling behaviour	-	FPCA	-	-	-	[77]
	Biomechanics	Kinematic gait data (knee flexion angle)	Cubic B-spline	FPCA	LDA	-	-	[78]
	Biomechanics	Knee joint kinematics in the vertical jump	B-spline	FPCA	-	-	-	[79]
	Biomechanics	Kinematic gait data (sit to stand movements)	B-spline	-	-	-	-	[80]
	Ecology	Water quality trend data (nutrient and sediment)	Fourier	FPCA	HCA	FRM	-	[81]
	IT	Software complexity measure	Smoothing spline	-	-	-	-	[82]
	Linguistics	Tongue tip velocity	B-spline	-	-	-	-	[83]
	Physiology	Blood lactate for running speed on a treadmill	Polynomial spline	FPCA	-	-	-	[84]
	Psychology	Tension judgement in music	B-spline	-	-	-	-	[85]
2005	Biology	Protein expression profiles	P-spline	FPCA	HCA	-	-	[86]
	Biomechanics	Joint angles describing the limb motion	Regression spline	FPCA	-	-	-	[87]
	Biomedicine	Functional magnetic resonance imaging data from 1.5-Tesla Magnetom vision	B-spline	FPCA	-	-	-	[88]
	Biomedicine	Functional magnetic resonance imaging data from 3.0 T Allegra system-	B-spline	FPCA	-	-	-	[89]
	Ecology	Smith McIntyre grab species	-	FPCA	HCA	-	-	[90]
	Education	Trends in Mathematics and Science Achievement (TIMSS) score	Nonparametric spline	FPCA	CART,KNN	-	-	[91]
	Finance	Cash flows in point of sale and ATM networks	Fourier	-	-	FANOVA	-	[92]
	Linguistics	Speech movement records	Wavelets	-	-	-	-	[93]
	Psychology	Tension judgement in music	B-spline	-	-	-	-	[94]
2004	Chemistry	Molecular weight distributions	B-spline	-	-	FRM	-	[95]
	Medicine	Esophageal bolus flow	-	-	-	-	-	[96]
	Meteorology	Biomarkers	Cubic spline	FPCA	-	-	-	[97]
	Neurology	Automated atlas-based head size normalization	-	-	-	-	-	[98]
	Psychology	Musical emotions and tension	B-spline	-	-	-	-	[99]
2003	Biomechanics	Digitized images of hand drawing curves generated by subjects treated with various facial preparation	Fourier	FPCA	-	FANOVA	-	[100]
	Biomedicine	Longitudinal plasma folate data	Weighted local polynomial spline	FPCA	-	-	-	[101]
2002	Agriculture	Lodging score for rice fields based on a digital overhead image	Fourier	-	-	FRM	-	[102]
	Biomedicine	Myocardial contractile function images	Cubic B-spline	FPCA	-	-	-	[103]
	Economics	Monthly nondurable goods production index	B-spline	-	-	-	-	[104]
	Medicine	Foetal heart rate data	Fourier	-	-	FRM	-	[18]
	Medicine	Foetal heart rate data	Fourier	-	-	FLRM	-	[19]
2001	Satellite	Radar electromagnetic signals	Kernel	FPCA	-		-	[105]
2000	Biomechanics	Handwriting in Chinese	B-spline	FPCA	EDO	-	-	[14]
	Linguistics	Harmonics-to-noise ratio of voice signals	B-spline	-	-	-	-	[106]
	Meteorology	Annual cycle of sea surface temperatures	Polynomial spline	-	-	-	FAR	[107]
1999	Linguistics	Harmonics-to-noise ratio of voice signals	-	-	-	-	-	[108]
1998	Ecology	Abundance of the gray-sided vole Clethrionomys rufocanus	Log-spline	FPCA	-	-	-	[109]
1996	Linguistics	Vocal tract lip motion during speech	Smoothing spline	FPCA	-	FANOVA	-	[110]
1995	Biomechanics	Records of the force exerted by pinching a force meter with the tips of the thumb and forefinger an opposite sides	-	FPCA	-	-	-	[111]
	Economics	Income distribution	-	FPCA	-	-	-	[112]

FPCA - Functional principal component analysis; FEM - Functional embedding; HCA - Hierarchical cluster analysis; SVM - Support vector machine; LDA- Linear discriminant analysis; QDA - Quadratic discriminant analysis; KNN- K-nearest neighbours; MBC - Model based clustering; CART - Classification and regression tree; EDO - Estimated differential operators; FLM - Functional linear model; FRM - Functional linear regression model; FANOVA - Functional ANOVA; FMANOVA - Functional MANOVA; FFT - Functional F test; FLRM - Functional logistic regression model; FAR - Functional auto regressive model.

The earliest identified application of FDA was in 1995 and 75% of the reviewed articles were published since 2005. This reflects increasing recognition of the important features of functional data and awareness of the development of new statistical approaches and software for handling them.

While diverse fields were covered in the published studies, almost 21% of the studies related specifically to biomedical science (18 identified papers), followed by biomechanics applications (11 papers). Other fields of application were medicine (10), linguistics (6), biology (4), ecology (4), psychology (4), meteorology (4), environmental studies (4), demography (3), finance (3), neurology (2), economics (2), engineering (2), agriculture (1), physiology (1), information technology (1), education (1), chemistry (1), geophysics (1), and behavioral science (1). In relation to specific health issues, the most common topics were analyses of kinematics gait data (9 papers), magnetic resonance imaging (6 papers), and yeast cell cycle temporal gene expression profiles (6 papers).

### Important features of the published FDA studies

Table 1 summarizes the published studies in terms of their use of the following key features of FDA: the reported smoothing technique, FPCA, clustering, the adopted forms of the FLM and forecasting. The importance of each of these features is explained below and an overview given of how they were handled in the published studies.

#### Smoothing techniques

Smoothing is the first step in any FDA, and its purpose is to convert raw discrete data points into a smoothly varying function. This emphasizes patterns in the data by minimizing short-term deviations due to observational errors, such as measurement errors or inherent system noise. When reporting FDA studies, it is important to state the smoothing approach used because observational errors always exist in longitudinal data.

Table 1 summarizes the various smoothing techniques used to estimate functions from the discrete observations reported in the reviewed literature. Overall, all except twelve of the studies (i.e. 85.7% of the reviewed studies) provided information about the type of smoothing technique used. Although some authors believe that FDA can be considered as a smoothed version of multivariate data analysis, smoothing techniques should still be used to reduce some of the inherent randomness in the observed data [1,25,113].

Overall, B-spline smoothing was the most popular smoothing technique used (25 papers), presumably because of its simplicity and flexibility for tackling a wide range of nonparametric and semiparametric modeling situations. A common approach towards B-spline smoothing is to construct a large number of knots (as the smoothing parameter) to reduce the effective degrees of freedom and increase smoothness in the overall function estimate [114,115]. Other smoothing techniques adopted in the published studies included use of Fourier smoothing (8 papers), regression splines (6), kernel smoothing (7), polynomial splines (5), cubic splines (3), smoothing splines (3), wavelet bases (3), roughness penalties (2), local polynomials (2), local quadratics (1), local weighted regression (1), P-splines (1) and log-splines (1).

Ramsay and Silverman [9] emphasize that the choice of smoothing technique is dependent upon the underlying behavior of the data being analyzed. Ideally, the smoother should reflect or have features that match those of the data. For example, Fourier smoothers are traditionally used when the data are cyclical or periodic. Environmental diurnal ozone and NO_x_ cycles [71,116], trends in ecologically meaningful water quality variates in ecology [81], cash flows in finance [92] and fetal heart rate monitoring in medicine [18,19] are examples of the application of Fourier smoothers. Splines (regression splines, polynomial splines, B-spline) are typically chosen to represent noncyclical nonperiodic data [25,51,84], and wavelet bases are chosen to represent data displaying discontinuities and/or rapid changes in behavior [117,118]. Most recently, Ullah and Finch [23] used constrained penalized regression splines with a monotonic constraint to represent their smooth curves of falls incidence rates.

#### Data reduction

The FPCA is one of the most popular multivariate analysis techniques for extracting information from functional data [119,120]. This approach reduces the dimensions of a data set in which there are a large number of interrelated variables, while still holding as much of the total variation as possible. This reduction is obtained by transforming the data to a new set of variables, or principal components, that are uncorrelated and ordered so that the first few retain most of the variation present in all of the original variables.

The use of FPCA was reported in 51 (60.7%) of the reviewed studies (Table 1). It has been successfully applied to real life problems such as modeling the curvature of the cornea in the human eye [11], in a set of density curves where the argument variable is log income [121], and fMRI scans of areas in the human brain [88]. Many different applications of principal component analysis to functional data have been developed, including a useful extension of FPCA that allows the estimation of harmonics from fragments of curves [122]. Although FPCA is an important feature of FDA, not all studies reported it because they did not undertake data reduction. For example, Roislin et al. [48] used a functional regression model to estimate the effects of gender, age, and walking speed on kinematic gait data; Park et al. [58] classified gene functions using a support vector machine (clustering) for time-course gene expression data; and Lucero [93] used only a B-spline to smooth the harmonics-to-noise ratio of voice signals. None of these applications required FPCA to reduce the data.

#### Clustering

While FPCA results in dimension reduction, FPCA vector scores can be used for clustering different functions/components using standard clustering methods. Clustering is one of the most frequently used techniques for partitioning a dataset into subgroups that contain instances that are similar to each other while being clearly dissimilar to those of other groups. In a functional context, clustering helps to identify representative curve patterns and individuals who are very likely to be involved in the same or similar processes. For example, in time-course microarray experiments, thousands of gene expression measures are taken over time [123] and an important problem is to discover functionally related genes that could then be the target for new gene regulatory networks or functional pathways. Clustering of data allows identification of groups of genes with similar expression patterns to identify such networks and pathways.

A number of clustering methods were reported in the reviewed literature (Table 1) and most of these were exploratory techniques for gene expression data. Overall, 15 studies (17.9%) reported some form of clustering. Biologists and ecologists used clustering to classify genes [68,76] and ecological components [81,90] within their studies. The most commonly applied clustering algorithms were hierarchical in nature (7 papers). Hierarchical algorithms define a dendrogram (tree) relating similar objects in the same sub-trees. In each step, similar sub-trees (clusters) are merged to form a dendrogram that clearly shows the different distinct clusters. Other reported clustering methods were linear discriminant analysis (LDA) (2 papers), k-nearest neighbors (KNN) (3), support vector machine (SVM) approaches (2), model-based clustering (MBC) (1), quadratic discriminant analysis (QDA) (1) and estimated differential operators (EDO) (1).

The LDA and QDA are both classical clustering methods and commonly used in microarray analysis [124,125]. Application of LDA is based on finding linear combinations of gene expression levels called discriminants that maximize the ratio of between-group variation to within-group variation. The QDA approach is a generalization of the linear classifier, allowing covariance matrices to be heterogeneous, whereas LDA functions are based on the assumption that covariance matrices of each of the classes are equal. This assumption relaxation can prevent individuals from being placed into classes with larger variance on their covariance matrix diagonals. The KNN is a nonparametric classification method based on the distance between individuals [126,127]. For example, Song et al. [59] proposed KNN to classify time-course gene expression profiles based on information from the data patterns. The SVM approach [128] is an extremely powerful methodology for classification problems and has a wide range of applications. Recently, this method has received much attention in classification problems that arise with the analysis of microarray data [58,59]. The MBC method assumes that the data are generated by a multivariate normal mixture distribution with appropriate means and covariance matrix [129]. Song et al. [68] have applied this method of clustering time-course gene expression data.

#### Functional linear models

An interesting application of FDA involves the construction of functional models that describe the relation between an outcome variable and an explanatory variable. Such models are termed functional linear models (FLMs). The number of published applications involving functional data has been steadily growing. In functional linear models, the functions could be the outcome or the predictors or both.

Of the reviewed studies in Table 1, 21 (25.0%) reported some form of FLM. The approach most favored by authors was a basic functional linear regression model (12 papers). When the outcome variable is in its functional form and the relationship is almost linear, the methodology is called functional linear regression model, or FRM. Functional ANOVA (FANOVA) was used in eight studies. Vines et al. [85] developed a functional F test (FFT) for linear models with functional outcomes in their psychological study for measuring tension judgment in music. They illustrated how to apply the FFT to longitudinal data where intrasubject repeated measures are viewed as discrete samples from an underlying curve with a continuous functional form. One study applied a functional logistic regression model (FLRM) to fetal heart rates [18] and another applied functional multivariate analysis of variance (FMANOVA) to temporal fertility trajectories of medfly populations [47].

#### Forecasting framework

The recent introduction of stochastic methods for forecasting functional data has significant advantages over the standard approaches for better understanding trends, risk factor relationships, and the effectiveness of preventive measures. A major advantage of these methods is that they can measure forecast uncertainty through the estimation of prediction intervals for future data. For this reason, the FDA forecasting approach has started to receive attention in both demographic and medical applications [24,25,28,60]. To date there has only been limited application of FDA to epidemiological studies relating to the prediction of incidence/prevalence rates, with only one recent study applying it to forecast the incidence of fall-related severe head injuries [23].

Overall, only seven of the reviewed studies (8.3%) reported any FDA-derived forecasting. A state space model was the most common approach for forecasting functional data in these studies (5 papers). In the forecasting process, the authors estimated the coefficients from a time series, with one value representing each time point, and a state space model was used to model and forecast these time series coefficients [23-25,28,130].

## Discussion

Modern data analysis has greatly benefited from the development of FDA methods and their application to time series data. Although used by statisticians for many years, FDA provides a relatively novel approach to modeling and prediction that is highly suitable for public health and biomedical applications. This paper has summarized papers describing FDA applications with a main emphasis on five popular features: smoothing, FPCA, clustering, FLM, and forecasting.

Overall, the published FDA application studies demonstrate the value of this approach for exploring complex multivariate functional relationships and its major strength of being able to model the functional form of time series data. Different approaches allow for FDA representations as smooth functions, and the published studies used a range of smoothing techniques for the estimation of discretely observed data. The FDA approach of initially smoothing the data and then using the smoothed observations for modeling and forecasting is a major methodological improvement over methods that simply fit linear/non-linear trends to observed data. These FDA approaches are very suitable for widespread public health and biomedical applications. Although some authors believe that FDA can be considered as a smoothed version of multivariate data analysis, recent work has shown the advantage of direct application of smoothing techniques to reduce some of the inherent randomness in the observed data [1,25,113].

The theoretical and practical developments that have occurred over recent years mean that researchers can now successfully apply FPCA to many practical problems, with main attention given to the reduction of data dimensions to a finite level and identification of the most significant components of the data. High dimensional data significantly slow down conventional statistical algorithms and in some cases it is not feasible to use them in practice. This means that standard classification methods can suffer from difficulties when handling such data. Some studies need to compress their data to facilitate exploration of the most important features (e.g., characteristics of genes from entire time-course data). In such instances, dimension reduction should be applied to keep only the relevant information and for removing correlations. This will both speed up and improve the accuracy of subsequent analyses and modeling. The FPCA has proven to be a key technique for dimension reduction, reported in most of studies reviewed here. It can also be used to investigate the variability of data with respect to individual curve shapes [131].

One of the major application areas highlighted in this review is an apparent increasing interest in clustering and classification techniques, especially for time-course gene expression data. The clustering is useful for detecting patterns and clusters in high dimensional functional data. Functional clustering is used to search for natural groupings of data with similar characteristics. Unlike conventional clustering that requires measuring multivariate data at the same time points to calculate Euclidean ‘distances’ between observations, functional clustering can derive a broader class of distance measures even if the original measurements are not time-aligned among sampling units, as is common in public health applications. The reason for the popularity of functional clustering is that it can classify time series data into different classes without requiring *a priori* knowledge of data.

A very interesting application of FDA involves the construction of linear models that describe the relation between an outcome variable and explanatory variables with functional nature. The FLMs have recently gained popularity and the related literature has been steadily growing with several studies using covariates to explain functional variables. Overall, FRM and FANOVA methods were the most prominent in the reviewed literature. Reasons for not using FLM techniques are unclear but might include a lack of knowledge about the value of building functional models for public health and biomedical data. However, the use of FLM is not always necessary and depends on the specific research questions.

Public health researchers now recognize the importance of understanding trends in high dimensional time series data. Policy makers, for example, need information about predicted trends to inform their decision-making about public health and economic investments to reduce the burden into the future [132]. It is critical that such predictions are robust and based on the best available statistical modeling approaches to minimize possible errors in the forecasts. This is also true for other areas of public health and biomedicine. The new FDA forecasting approaches [23,25] are a natural extension of methods developed for mortality and fertility forecasting that have evolved over the last two decades in demography [25,133,134]. The methodology has therefore been used in a number of demographic applications and there have been various extensions and modifications proposed [25,134]. Somewhat surprisingly, the use of FDA forecasting in public health and biomedical applications has been limited to date.

## Conclusion

In summary, this paper describes FDA and its important features as applied to time series data from various fields. Functional data analysis provides a relatively novel modeling and prediction approach, with the potential for many significant applications across a range of public health and biomedical applications. Importantly, not all FDA features always need to be used in a single study and the selection of specific analysis features will depend on the underlying behavior of the data, the nature of study and the specific research questions being posed. Consideration should be given to wider application of FDA and its important modeling features so that more accurate estimates for public health and biomedical applications can be generated.

## Abbreviations

FDA: Functional data analysis; FPCA: Functional principal component analysis; FLM: Functional linear modeling; LDA: Linear discriminant analysis; KNN: K-nearest neighbours; SVM: Support vector machine; MBC: Model based clustering; QDA: Quadratic discriminant analysis; EDO: Estimated differential operators; FRM: Functional linear regression model; FANOVA: Functional ANOVA; FFT: Functional F-test; FLRM: Functional logistic regression model; FMANOVA: Functional multivariate analysis of variance.

## Competing interests

The authors have no conflicts of interest that are directly relevant to the content of this review.

## Authors’ contributions

As first author, SU conceived and designed the study reported in this paper. He took the lead role in drafting the manuscript and reviewed all relevant articles and analysed their content. The second author, CF, provided expertise in the conduct of systematic reviews and also contributed to the writing and editing of the paper. She reviewed the article and revised it critically for important intellectual content. Both authors read and approved the final manuscript.

## Pre-publication history

The pre-publication history for this paper can be accessed here:

http://www.biomedcentral.com/1471-2288/13/43/prepub
